# Unexplained Positional Hypoxemia in an Elderly Patient: A Case of Platypnea–Orthodeoxia Syndrome

**DOI:** 10.7759/cureus.105715

**Published:** 2026-03-23

**Authors:** María Alejandra Villalobos De Ycaza, Giva Mariel López Saborio, Jean Jaasiel Mora Murillo, Esteban Alvarado Sanabria, Mónica Quirós Alvarado, Kendal Leandro Sandí

**Affiliations:** 1 General Medicine, Faculty of Medicine, University of Costa Rica, San José, CRI

**Keywords:** elderly patient, migraine, orthodeoxia, patent foramen ovale, platypnea

## Abstract

Patent foramen ovale is a congenital cardiac condition that may remain clinically silent until advanced age. One of its potential manifestations is platypnea-orthodeoxia syndrome, characterized by hypoxemia in the upright position with improvement in the supine position. Diagnosis may be delayed and challenging in older adults, as hypoxemia is frequently attributed to alternative conditions such as infection, pulmonary disease, or age-related physiological changes.

We report the case of an 88-year-old man admitted with suspected urosepsis in whom refractory hypoxemia of unclear origin was documented despite unremarkable pulmonary and initial cardiac imaging studies. Marked oxygen desaturation occurred when seated or standing and resolved in the supine position. Two-dimensional transesophageal echocardiography with bubble study confirmed the presence of a patent foramen ovale with right-to-left shunting. Percutaneous closure was successfully performed, resulting in complete resolution of hypoxemia. Notably, the patient also experienced a reduction in the frequency and intensity of migraine episodes following the intervention.

To our knowledge, reports of symptomatic platypnea-orthodeoxia syndrome requiring percutaneous closure in patients of such advanced age remain limited. This case emphasizes the importance of considering intracardiac shunting in older patients with unexplained positional hypoxemia and supports the role of individualized decision-making regarding patent foramen ovale closure to preserve functional status in selected elderly individuals.

## Introduction

All mammals have a patent foramen ovale during the fetal stage [1]. The formation of the foramen ovale and both atria begins at four weeks of gestation [1]. The foramen allows a shunt of oxygenated blood from the right atrium to the left atrium [2]. At birth, the decrease in pulmonary and right atrial pressure, along with the expulsion of amniotic fluid from the lungs, results in the pressure in the left atrium exceeding that of the right atrium [1]. This induces the closure of the septum primum over the septum secundum [1]. In four out of five adults, the foramen ovale becomes closed; however, in 20-25% of individuals, complete fusion of these septa does not occur, so the foramen ovale remains patent [1]. In this latter group, clinical manifestations are usually absent because the volume load on the right ventricle is not sufficient to cause structural changes; however, it may be associated with platypnea-orthodeoxia, emboli, acute myocardial infarction, and decompression sickness [1].

This issue is particularly relevant in older adults, as the clinical characteristics of cryptogenic infarction in this population remain poorly understood, given that previous studies have primarily focused on individuals under 60 years of age [3]. Furthermore, current guidelines recommend closure of the patent foramen ovale as a secondary prevention measure mainly in this non-geriatric age group; however, performing this procedure may also be appropriate in older patients depending on the clinical circumstances [2,4].

This report presents the case of an older adult diagnosed with a patent foramen ovale, highlighting the diagnostic challenges and therapeutic considerations in this age group.

## Case presentation

An 88-year-old Panamanian male with a history of hypertension, atrial fibrillation, migraine with aura, and benign prostatic hyperplasia, on treatment with warfarin and losartan, and with a permanent Foley catheter in place, sought medical attention due to a feverish sensation. He has a history of recurrent urinary tract infections. One month before the current episode, a urine culture grew extended-spectrum beta-lactamases (ESBL) -negative Klebsiella pneumoniae. 

At presentation, the patient was found to be hypoxemic, with oxygen saturation of 84% on room air. The initial diagnostic impression was acute hypoxemic respiratory failure with suspected sepsis. He was admitted to the intensive care unit, started on meropenem, and placed on high-flow nasal cannula. 

Initial physical examination revealed rhythmic heart sounds without audible murmurs, no added respiratory sounds, and no other significant abnormalities. However, a subsequent examination documented a grade 2/6 diastolic murmur at the aortic focus. 

Laboratory studies showed elevated troponin I, normal creatine kinase-myocardial band (CK-MB), elevated N-terminal pro-B-type natriuretic peptide (NT-proBNP), and normal D-dimer levels. Transthoracic echocardiography performed on admission revealed a left ventricle of normal size and volume with severe concentric hypertrophy and preserved systolic function (left ventricular ejection fraction 62%). The left atrium was moderately dilated. The right-sided chambers were normal in size and function. Mild aortic and tricuspid regurgitation were noted, with an estimated pulmonary artery systolic pressure of 25 mmHg. The ascending aorta measured 4.7 cm. Additionally, the inferior vena cava was not dilated and showed greater than 50% inspiratory collapse. No pericardial effusion was observed. 

Electrocardiography demonstrated sinus rhythm, poor R-wave progression in the precordial leads, without ST-elevation or T-wave changes. Despite elevated troponin levels, neither echocardiogram nor electrocardiographic findings suggested myocardial ischemia.

Broad-spectrum antibiotics were continued while awaiting culture results. Clinically, the patient improved, with decreasing leukocytosis, negative procalcitonin, and resolution of fever.

On the following day, he developed increased work of breathing, with oxygen saturation dropping to 78% despite a high-flow nasal cannula. Point-of-care ultrasound (POCUS) revealed no pleural effusion, no B-lines, and preserved myocardial contractility. Co

**Figure 1:**
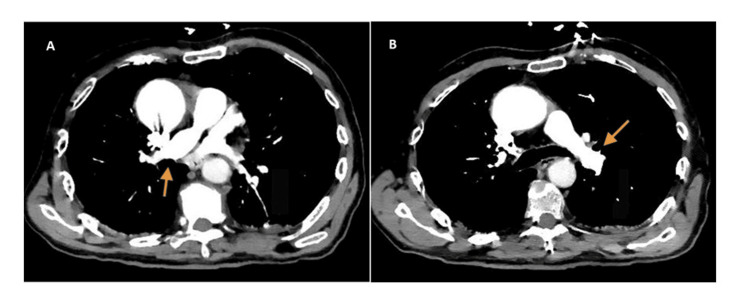
Chest computed tomography (CT) pulmonary angiography. A. The orange arrow indicates the right pulmonary artery branch. B. The orange arrow indicates the left pulmonary artery branch. No evidence of pulmonary thromboembolism is observed.

Computed tomography (CT) pulmonary angiography was negative for pulmonary embolism (Figure 1).

**Figure 2:**
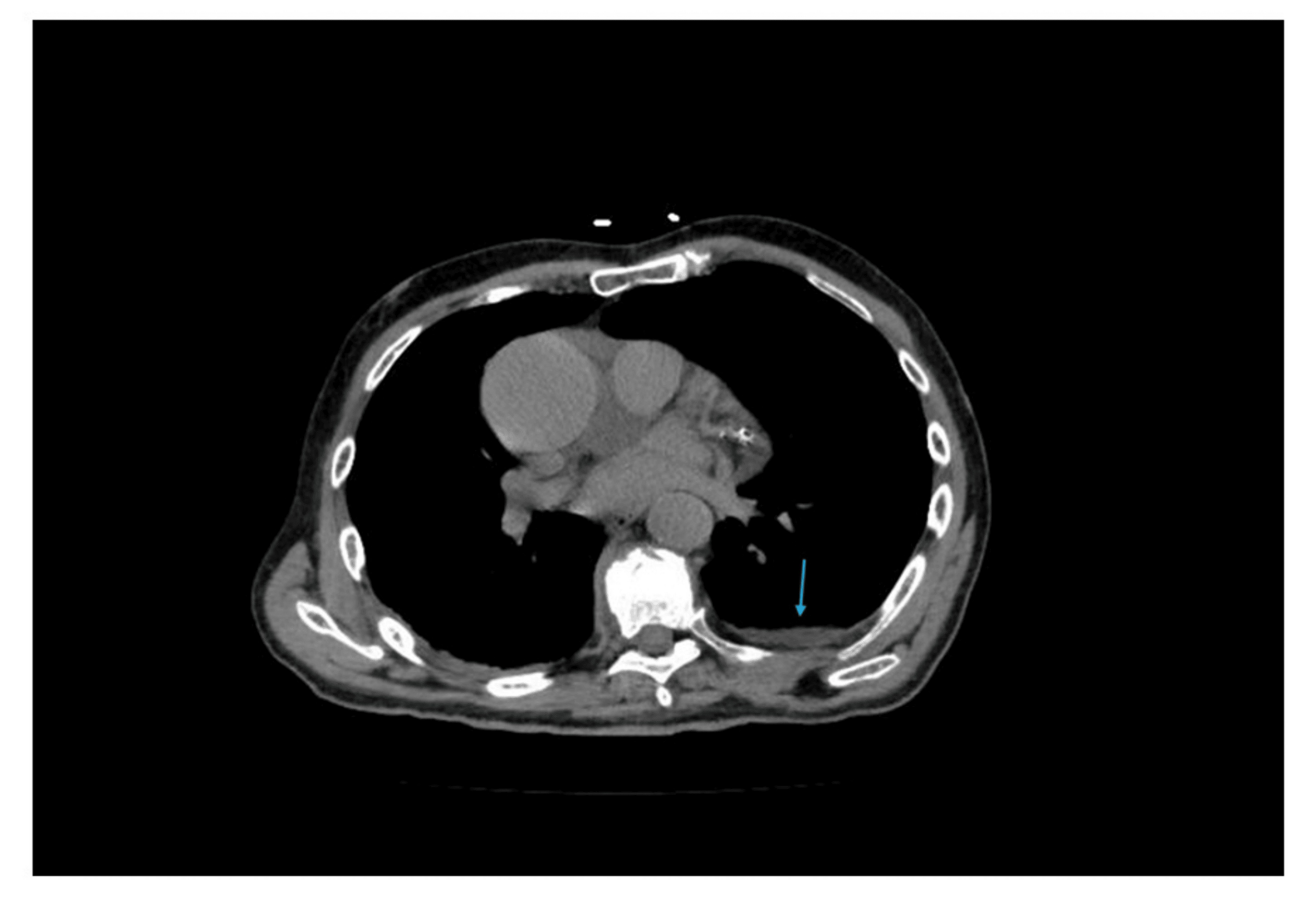
Chest computed tomography (CT) demonstrating left pleural effusion (light blue arrow).

Forty-eight hours later, physical examination revealed discrete crackles at the left lung base without peripheral edema. Noninvasive positive pressure ventilation was attempted but not tolerated, so the patient remained on high-flow nasal cannula. Repeat POCUS revealed posterior B-lines and a small left pleural effusion without significant atelectasis; no femoral thrombi were identified. A subsequent high-resolution chest CT showed minimal unilateral pleural effusion without other significant findings (Figure 2).

After seven days of hospitalization, he was transitioned to a standard nasal cannula at 5 liters per minute (L/min), with oxygen saturation ranging from 85% to 95%. He also complained of discomfort in the left lower limb without edema, temperature changes, or catheter-related issues. The prescribed course of meropenem was completed on day eight. 

Up to this point, the patient had only reported mild, occasional dyspnea. During hospitalization, he received physical therapy and was instructed to sit up twice daily. However, the patient reported dyspnea and oxygen desaturation upon assuming the upright position.

On day nine, the patient reported numbness and weakness in the left lower limb. The same day, oxygen saturation dropped below 70% during physical therapy in the seated position. Additional episodes were documented in which oxygen saturation decreased to 80% while seated, even without exertion, with recovery to 97% when supine, consistent with positional hypoxemia with a partial pressure of oxygen below 80 mmHg on arterial blood gas analysis.

Follow-up laboratory tests showed a progressive decrease in troponin levels and improvement in inflammatory markers. 

Coronary computed tomography for calcium scoring revealed right coronary dominance, high implantation of the right coronary ostium, and patent left coronary ostia with no significant atherosclerotic disease. The aortic root measured 51 mm, and the ascending aorta 53 mm.

After 16 days of hospitalization, postured-dependent oxygen desaturation persisted with or without supplemental oxygen, despite clinical improvement of the infectious process. The patient remained on a nasal cannula at 5 L/min. Initially, this was attributed to sepsis-related pulmonary changes. On day 17, a decision was made to perform another echocardiogram, this time with saline bubble injection.

Regarding the patient’s complaint involving the left lower limb, it was determined that he developed traumatic neuropathy associated with the placement of compression bandaging for thrombosis prevention. Neurological evaluation revealed paresthesia of the left foot during dorsiflexion and 3/5 strength in foot abduction, which impaired proper ambulation. Injury to the left common peroneal nerve was suspected. Electrophysiological studies demonstrated F-wave abnormalities suggestive of lumbosacral radiculopathy, along with distal sensory neuropathy, with preserved motor conduction velocities in the tibial and peroneal nerves.

Notably, during several episodes of oxygen saturations of 83-84%, and even as low as 60% during physical therapy, the patient remained asymptomatic.

**Figure 3:**
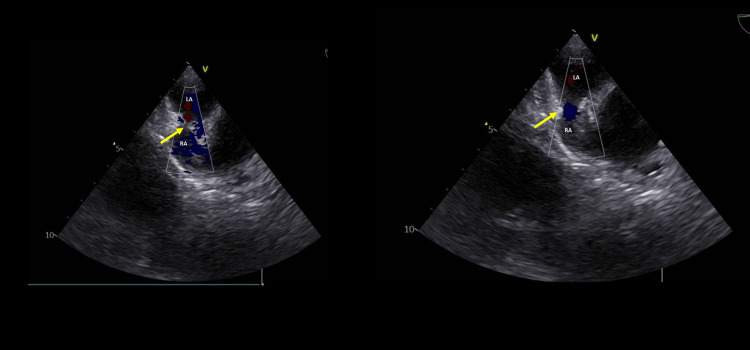
Two-dimensional Doppler echocardiogram. RA: right atrium. LA: left atrium. The yellow arrow indicates blood flow (color Doppler) through the patent foramen ovale.

**Figure 4:**
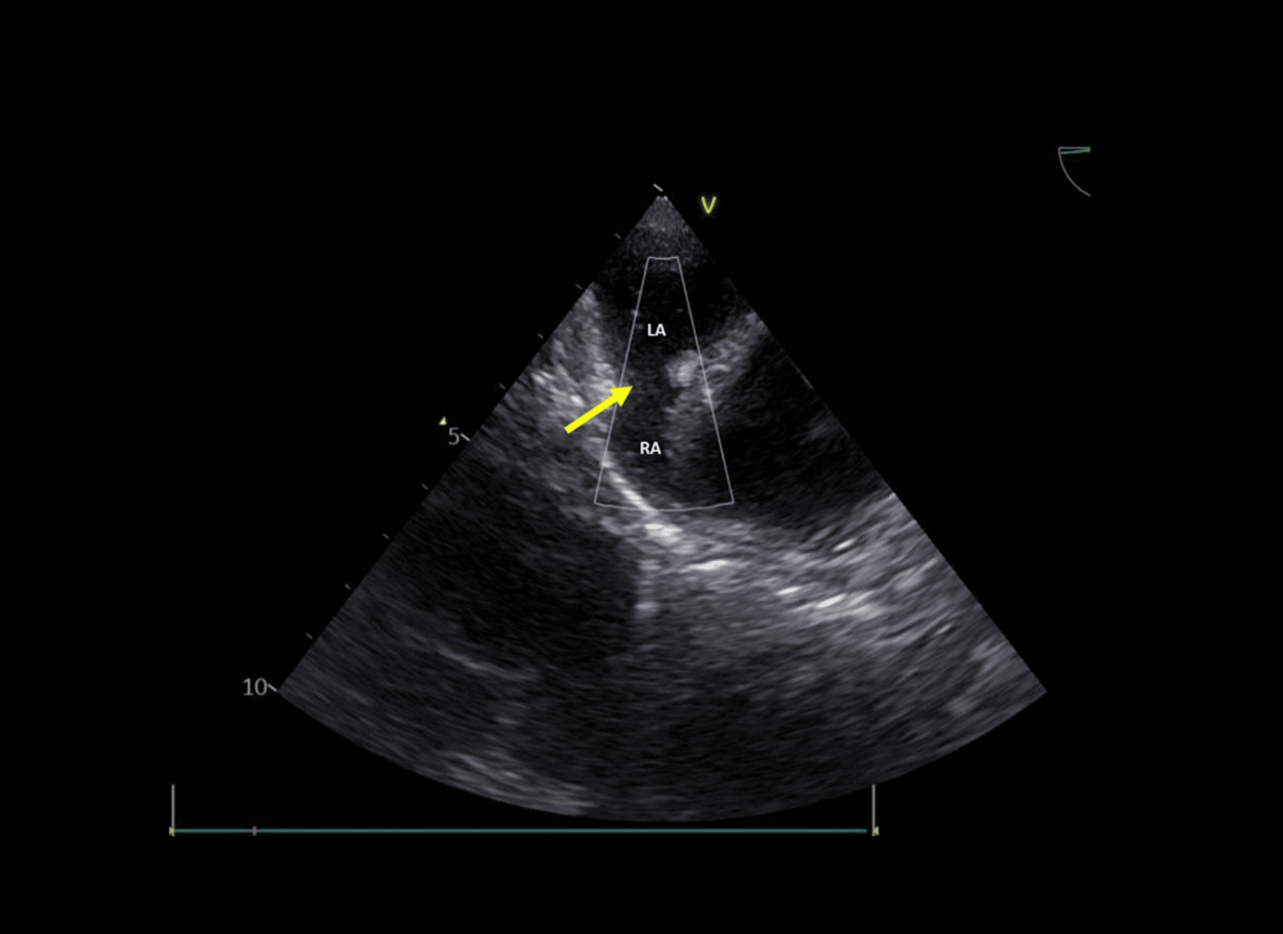
Two-dimensional echocardiogram. RA: right atrium. LA: left atrium. The yellow arrow indicates the patent foramen ovale.

**Figure 5:**
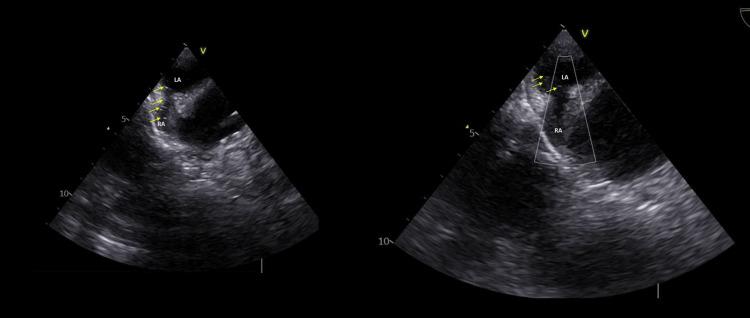
Two-dimensional echocardiogram with agitated saline contrast. The yellow arrows indicate the passage of agitated saline microbubbles across the patent foramen ovale.

On day 27, transesophageal echocardiography with bubble study demonstrated a patent foramen ovale, normal biventricular function, and annuloaortic ectasia (Figures 3, 4, 5).

Regarding the initial infection, the patient remained afebrile during hospitalization. Urine culture grew Serratia species sensitive to ceftriaxone.

Repeat neurological examination after several sessions of physical therapy showed 5/5 proximal lower limbs strength, 5/5 strength in the right foot, and 4/5 strength in the left foot with mild paresis of hallux extension. Deep tendon reflexes were not hyperactive; Hoffmann’s sign was negative. Patellar and Achilles reflexes were brisk, predominantly on the left, without ankle clonus or Babinski sign. Physical therapy was discontinued due to recurrent desaturation episodes as low as 56%. 

Given the diagnosis of patent foramen ovale associated with positional hypoxemia, percutaneous closure was indicated. On day 42 of hospitalization, transcatheter closure of the patent foramen ovale was successfully performed. Following the procedure, oxygen saturation improved, and he required only 2 L/min via nasal cannula. Two days later, he tolerated physical therapy without desaturation. By postoperative day three, oxygen saturation was 96-98% on room air.

Taking into account the favorable cardiac outcome and his history of recurrent urinary tract infections, including the episode of sepsis that prompted admission, the patient subsequently underwent cystoscopy and transurethral resection of the prostate without complications. Although he initially failed to void spontaneously, he was discharged three days later. Four days after discharge, he achieved spontaneous and clear urination.

## Discussion

The case described involves a patient with refractory hypoxemia in whom multiple diagnostic studies yielded inconclusive results. CT pulmonary angiography ruled out pulmonary embolism, high-resolution chest CT revealed no significant findings, and the initial echocardiogram showed no evidence of significant pulmonary hypertension. However, despite this apparently confusing clinical presentation, the patient had a clear platypnea-orthodeoxia syndrome. Platypnea-orthodeoxia syndrome is defined as normal oxygen saturation in the supine position with a decrease below 92% when the patient is seated or standing [1].

Given the markedly reduced oxygen saturation levels, reaching as low as 56% while seated, the question arises as to why the diagnosis was challenging. Initially, hypoxemia was presumed to be secondary to sepsis, atelectasis, alveolar changes, or even advanced age. Factors contributing to delayed or incorrect diagnosis of platypnea-orthodeoxia syndrome in older adults have been described, including the normalization of dyspnea as a “common” symptom in this population or attribution of symptoms to other cardiovascular risk factors [5]. Additionally, oxygen saturation is typically measured with the patient in the supine position during routine clinical evaluation [5]. Also, limited physician awareness of this syndrome and its management may also contribute to delayed diagnosis [5]. The gold standard for diagnosing patent foramen ovale is transesophageal echocardiography with bubble study [3]. Recent studies indicate that three-dimensional (3D) transesophageal echocardiography is more precise than two-dimensional imaging; however, the disadvantages of 3D imaging include operator dependency, the need for adequate Valsalva maneuver, and lower image resolution [6]. In this patient, although orthodeoxia had already been clearly documented, transesophageal echocardiography (2D) with bubbles was performed late in the clinical course.

The delayed clinical manifestation of patent foramen ovale may also represent a diagnostic enigma. Although present from birth, it may remain asymptomatic until advanced age, as observed in this case. Notably, the patient remained without symptoms despite significant desaturation, suggesting possible chronic physiological adaptation. Thoracoabdominal surgeries and dilation of the ascending aorta have been associated with clinical presentations similar to those described in this case [5]. It has been proposed that such anatomical distortion alters cardiac structure and increases right-to-left shunting through the patent foramen ovale, likely due to changes in interatrial septal position and septal orientation [5,7]. Furthermore, blood flow from the inferior vena cava to the right atrium is more likely to be directed toward the ostium secundum portion of the septum [8]. Additionally, a connective tissue disorder has been hypothesized in patients with patent foramen ovale and aortic dilation [8]. In the present case, the patient had dilation of both the aortic root and ascending aorta.

The association between migraine and patent foramen ovale has been described since the early 1900s, when the relationship between migraine with aura and thromboembolic cerebrovascular events was first explored [9]. Subsequent studies demonstrated an independent association between ischemic events and migraine; moreover, multiple meta-analyses have shown an increased prevalence of patent foramen ovale in patients with migraine [9]. The degree of right-to-left shunting is greater in cases of migraine with aura [5]. One proposed mechanism involves vasoactive substances in venous blood that would normally be metabolized in the pulmonary circulation [5]. In the presence of a patent foramen ovale, these substances bypass pulmonary filtration and reach the intracranial circulation, potentially triggering migraine episodes [5]. Serotonin released through platelet activation is one of the substances implicated in this hypothesis [5]. Although closure of a patent foramen ovale may improve migraine symptoms in certain patients, this effect is not universal [5]. Most experts agree that an association exists between patent foramen ovale and migraine, although there is limited consensus regarding the precise physiological link [9]. In the present case, following closure of the patent foramen ovale, the patient experienced improvement in migraine episodes, with reduced frequency and intensity.

Regarding management, the Society for Cardiovascular Angiography & Interventions guidelines recommend closure of the patent foramen ovale in patients who have experienced a cerebrovascular event associated with the defect or who present with platypnea-orthodeoxia syndrome [10]. Routine closure in individuals older than 60 years remains controversial, in contrast to younger adults with ischemic events related to the defect, in whom closure is considered relatively safe and effective in preventing recurrence [4]. Despite this, studies have demonstrated that patent foramen ovale closure is safe and effective in the long term in patients over 60 years of age [10]. Relatively few adverse events have been reported, with only a slight increase in atrial fibrillation compared to patients younger than 60 years [11]. The decision to perform closure should consider the patient’s functional status [4]. In this case, before hospitalization, the patient was independent in basic activities of daily living; therefore, the implications of not performing closure, including the potential need for indefinite supplemental oxygen therapy, were carefully considered. The patient in this report underwent closure without complications.

An additional element in this case was the development of left lower limb neuropathy during hospitalization. One differential consideration was embolism associated with the patent foramen ovale; however, the neurological deficit was characterized as peripheral rather than central. Nevertheless, this condition significantly affected the functional status of the older adult, underscoring the importance of comprehensive and multidisciplinary management during hospitalization.

## Conclusions

Platypnea-orthodeoxia syndrome remains an underrecognized cause of refractory hypoxemia in older adults. This case underscores the importance of assessing oxygen saturation in different postures and maintaining a high index of suspicion for intracardiac shunting when conventional diagnostic studies are inconclusive. In selected elderly patients with preserved functional status, transcatheter closure of patent foramen ovale can be safe and clinically beneficial, even beyond the age groups traditionally emphasized in guidelines.
